# Facilitating conditions for staff’s confidence to enforce school tobacco policies: qualitative analysis from seven European cities

**DOI:** 10.1186/s43058-022-00362-7

**Published:** 2022-10-22

**Authors:** Anu Linnansaari, Michael Schreuders, Anton E. Kunst, SILNE-R -study group, Arja Rimpelä, Jaana M Kinnunen, Vincent Lorant, Adeline Grard, Nora Mélard, Pierre- Olivier Robert, Matthias Richter, Martin Mlinarić, Laura Hoffman, Luke Clancy, Sheila Keogan, Elisabeth Breslin, Joan Hanafin, Bruno Federico, Diego Marandola, Anna di Marco, Paulien Nuyts, Mirte Kuipers, Julian Perelman, Teresa Leão, Joana Alves, Pirjo Lindfors

**Affiliations:** 1 Faculty of Social Sciences, Unit of Health Sciences, Tampere University, P.O. Box 100, 33014 Tampere, Finland; 2 Department of Public Administration and Sociology, Erasmus School of Behavioral and Social Sciences, Erasmus University Rotterdam, 3000 Rotterdam, DR The Netherlands; 3 Department of Public and Occupational Health, Amsterdam UMC, University of Amsterdam, Amsterdam Public Health Institute, Amsterdam, The Netherlands

**Keywords:** School tobacco policies, Smoke-free school policies, Implementation, Policy implementation, Tobacco prevention, Health promotion, Smoke-free school, Tobacco-free school, Tobacco-free environments

## Abstract

**Background:**

School staff members’ consistent enforcement of school tobacco policies (STPs) is needed to decrease adolescent smoking and exposure to tobacco smoke. Staff’s confidence, indicating their perceived ability to cope with students’ negative responses, explains variations in staff’s STPs enforcement, yet understanding of the determinants for confidence is lacking. We analyzed the conditions in which the staff feel confident in addressing students who violate STPs to support staff’s enforcement.

**Methods:**

Data consists of 81 semi-structured interviews with the staff members from 26 secondary schools in seven European cities in Belgium, Finland, Germany, Ireland, Italy, The Netherlands, and Portugal. In every city, 3–4 staff members (senior management, teachers, supportive staff) in 3–4 schools (academic–vocational, high–low SES area) were interviewed. Transcripts were analyzed with thematic analysis.

**Results:**

When staff felt confident in their ability to prevent, diminish, or handle students’ negative responses, they were more likely to address students on STP violations. The staff was more confident (1) when consistent policy enforcement within school and regarding the wider society ensured staff legitimacy for STPs enforcement, (2) when dialog and mutual familiarity with students allowed the staff to facilitate constructive interaction with STP violators, and (3) when organizational backup structures provided staff collegial support to overcome challenges in the enforcement. These conditions would support consistent enforcement, especially with persistent misbehavers and among the more uncertain staff members.

**Conclusions:**

Our study stresses the need to implement strategies at multiple levels to strengthen staff’s confidence for STP enforcement. To support staff’s legitimacy for enforcement, we suggest reinforcing structures and practices that facilitate consistency in STP enforcement; to support staff’s ability for constructive interaction with STP violators, we suggest strengthening staff’s social and emotional learning; and to support staff’s experience of collegial support, we suggest reinforcing staff’s collective ability to cope with students’ negative responses.

**Supplementary Information:**

The online version contains supplementary material available at 10.1186/s43058-022-00362-7.

Contributions to the literature

- The cross-country data generated and analyzed in this study allowed us to capture three conditions facilitating school staff’s confidence to enforce school tobacco policies (STPs). These conditions consider the complexity of real-world policy implementation.
- Our study responds to the dearth of evidence on the determinants influencing school staff’s enforcement of STPs, which is demonstrated to determine the effectiveness of STPs in decreasing adolescent smoking.
- Our findings allow us to provide multilevel strategies for schools and decision-makers to support staff’s confidence and consistent enforcement of STPs towards tobacco-free schools.

## Background

In 2021, Europe’s Beating Cancer Plan set the goal of the Tobacco-free Generation in Europe by 2040 [1]. Tobacco prevention is key to achieve this goal. Preventive policies are integral to the provisions of the WHO Framework Convention of Tobacco Control (WHO FCTC), which aims to protect present and future generations from the various health, social, environmental, and economic consequences of tobacco consumption, nicotine addiction, and exposure to tobacco smoke [2]. Schools represent an important setting for preventive efforts, as most tobacco users worldwide start smoking between 15 and 20 [3]. Aligned with the WHO FCTC Article 8 on Protection from exposure to tobacco smoke [2], school tobacco policies (STPs) are widely implemented in European countries [4–6]. STPs describe where, when, and for whom the prohibitions against using tobacco products—cigarettes in this study primarily—apply and the consequences for violating the rules.

Despite the wide implementation of STPs, evidence of their effectiveness in decreasing adolescent smoking is inconclusive [7, 8]. Galanti et al. [8] published a systematic review aiming to explain why studies about the effectiveness of STPs report such varying findings. They concluded that scholars have too often overlooked the exact mode of implementation and identified various elements of implementation that seem important for STPs to be effective. One of these elements is consistent enforcement of the rules by school staff on adolescents who violate the STPs [8–12]. Later research explained that consistency is vital because inconsistencies may cause adolescents to use loopholes in the enforcement as opportunities to smoke, develop pro-smoking social meanings around breaking the rules, decrease adolescents’ acceptance of the school authority over their smoking, and perceive the sanctions associated with violating the smoking rules as unjust and applied in a biased fashion [13].

Linnansaari, Schreuders, Kunst, Rimpelä, and Lindfors [14] utilized a realist review to explain the variety in school staff members’ enforcement of STPs, describing how the interconnections between STPs and contextual factors may explain staff’s enforcement through cognitive and psychosocial processes called mechanisms. Linnansaari et al. [14] identified several mechanisms that explain how the differences in the staff’s enforcement practices may originate. One mechanism is that some staff members may feel more confident than others in addressing students who violate the rules. This confidence depends considerably on staff members’ perceived ability—such as skills and authority—to deal with adolescents’ negative responses, like questioning the rules, showing disrespectful behavior, or refusing to comply with the staff’s instructions [14].

In their review, Linnansaari et al. [14] further emphasized the role of contextual factors in a staff’s confidence in STP enforcement, suggesting that specific factors at the societal, school, social, and individual may influence a staff’s perceived ability. For instance, national legislation prohibiting smoking in public places and at schools generated a sound basis for STPs, increasing a staff’s authority for enforcement, whereas a staff’s smoking status undermined this authority by providing students a means to question their authority. The staff also experienced uncertainties when they were unfamiliar with the students or when students’ characteristics prompted expectations of strong negative responses. However, Linnansaari et al. [14] stressed that limited empirical evidence shows which factors are important and how they may influence a staff’s confidence in enforcing STPs.

The role of confidence in a school staff’s behavior may also be reflected via knowledge of self-efficacy. Like confidence, self-efficacy indicates individuals’ beliefs in their capability to carry out actions or tasks [15, 16]. A large amount of empirical evidence shows that self-efficacy is one key factor in predicting and explaining individuals’ behavior [16]. Self-efficacy is also a core determinant in many behavioral theories [17], and essential for explaining teachers’ practices (e.g., [18–20]). For instance, a recent systematic review showed that teachers with higher self-efficacy intervened in bullying more often than teachers with lower self-efficacy [21]. Four sources are demonstrated to influence self-efficacy: having successfully performed the task previously, learning vicariously from observing others’ successful performances, being persuaded that one can perform the task, and reducing negative physiological and affective states associated with hesitation. However, little research has been done to explain determinants for self-efficacy; thus, a comprehensive understanding of its development is still lacking [16].

Considering the context is important to understand the school staff members’ confidence in STP enforcement and is a novel practice of real-world implementation studies characterized by complexity [22, 23]. We considered schools as Social Complex Adaptive Systems (SCAS), which aligns with Keshavarz [24]. SCAS indicates that the interactions among various adapting actors, such as senior management, teaching and supportive staff, students, and parents, fundamentally shapes the functioning of schools. These individual agents base their everyday actions on rules, including the formal organizational rules, such as school policies and guidelines, more abstract school ethos, and more general prevailing social norms and practices. Schools have some autonomy, yet they are also in a network of systems imposing multiple constraints. These external systems may be bigger than the school, such as the national education system, or smaller, such as families [24]. Understanding how a staff’s confidence emerges in the interplay within and among these individual, social, organizational, and societal systems may facilitate designing implementation strategies that support staff’s confidence.

We respond to the accounts of earlier research on the lack of evidence on determinants for staff’s confidence in STP enforcement. We aim to empirically study the conditions in which the staff feel confident in their ability to cope with students’ negative responses to STP enforcement. We consider the complexity of real-world policy implementation by recognizing and treating schools as SCAS, which indicates that we expect staff confidence to emerge in the interplay between various determinants at multiple levels. Our ultimate objective is to provide strategies for schools and decision-makers to support staff members’ consistent STP enforcement towards tobacco-free schools. The research questions for the study are as follows:

1. What is the challenge of STPs enforcement? Why does confidence matter in STPs enforcement?
2. In which conditions does the school staff feel confident in their ability to cope with students’ negative responses during STPs enforcement?

## Methods

Our report follows the Standards for Reporting Qualitative Research (SRQR) [25] (Additional file 1). The study employs interview data generated with 81 school staff members in 26 secondary schools in seven European cities: Namur (Belgium), Tampere (Finland), Hanover (Germany), Dublin (Ireland), Latina (Italy), Amersfoort (the Netherlands), and Coimbra (Portugal). The study was conducted as part of SILNE-R research project “Enhancing the effectiveness of programs and strategies to prevent smoking by adolescents: a realist evaluation comparing seven European countries” that was funded by Horizon 2020 between October 2015 and October 2018 [26]. The countries in the study were included as they represent great diversity in the implementation of national tobacco control policies and strategies. Six of the seven countries/cities were included as they were already part of the SILNE school survey (2012–2013). Ireland was added to the original countries due to its advanced tobacco control. The included cities were all median-sized and close to the national average in terms of socioeconomic level and percentage of non-foreign population.

From each country, three to four schools that represent different school tracks (academic–vocational) or were situated in areas with distinct socioeconomic levels were included in the study. Schools were generally recruited by contacting senior management of those schools that had participated already in the SILNE school survey, by providing information on the study and asking for participation. Overall, schools were willing to participate. An overview of the schools’ rules and countries’ legislation is presented in Table 1.

**Table 1:** Overview of governmental law on STPs, student smoking, and school rules on smoking (modified from [27, 28])

Country	Government law in 2016: prohibition on students and staff smoking in schools	School	Student weekly smoking^a^, %	STPs in 2016: which students are not officially prohibited from smoking during school hours, and where^b^
BEL	No smoking in the school buildings and on the premises	1	8.2	4th graders (avg. 15–16 years old) and above, outside the premises
		2	23	4th graders (avg. 15–16 years old) and above, outside the premises
		3	14.3	Any student with parental permission to leave for lunch, outside the premises
		4	6.2	4th graders (avg. 15–16 years old) and above with parental permission to leave for lunch, outside the premises
FIN	No smoking in the school buildings and on the premises	1	8.4	No smoking during school hours
		2	8.3	No smoking during school hours
		3	5.6	No smoking during school hours
		4	2.4	No smoking during school hours
GER	No smoking in the school buildings and on the premises	1	8.8	No smoking during school hours
		2	3.4	No smoking during school hours
		3	4.2	No smoking during school hours
IRL	No smoking in the school buildings and on the premises	1	1.8	No smoking while in school uniform
		2	4.9	No smoking while in school uniform
		3	2.6	No smoking while in school uniform
		4	8.9	No smoking while in school uniform
ITA	No smoking in the school buildings and on the premises	1	15.0	No smoking during school hours^c^
		2	26.5	No smoking during school hours
		3	10.8	Any student in a designated area on the premises
		4	44.6	No smoking during school hours
NDL	No smoking in the school buildings, except for ventilated “smoking rooms” (no prohibition on smoking on the premises)	1	6.6	3rd graders (avg. 14–15 years old) and above, outside the premises
		2	7.0	3rd graders (avg. 14–15 years old) and above, in a designated area on the premises
		3	21.5	3rd graders (avg. 14–15 years old) and above, outside the premises
		4	18.8	4th graders (avg. 15–16 years old) and above, in a designated area on the premises
POR	No smoking in the school buildings and on the premises	1	17.6	10th graders (avg. 15–16 years old) and above, outside the premises
		2	11.5	10th graders (avg. 15–16 years old) and above, outside the premises
		3	10.4	10th graders (avg. 15–16 years old) and above, outside the premises

^a^Questionnaires were completed by adolescents at school in the two grades enrolling students 14–16 years of age

^b^Detailed data on staff smoking bans in different schools are unavailable

^c^In Latina (ITA), most schools have comprehensive official rules, but their actual implementation is problematic. One school deliberately chose not to follow the law prohibiting smoking on the school premises

Three to four interviews in every school were conducted. The interviewees represented varying professional positions, including senior management, teachers, and supportive staff (e.g., receptionists, janitors, educators). Interviewees were recruited by the senior management or other school contact person who had the ability to select staff from varying positions and with comprehensive knowledge of STP enforcement. An overview of the interviewees’ country, school number, professional position, and age group, are presented in Additional file 2. This file also shows the code for each participant (e.g., BEL1S)—used in the “Results” section. The code indicates the participant’s country (Belgium), school number [1], and professional position (supportive staff).

The interview topic guide (Additional file 3) was formulated in collaboration with the research teams from all countries participating in the SILNE-R research project [26]. The Finnish researchers (AL, PL) coordinated the collaboration. With a professional teaching background, AL was familiar with the school context. The topic guide was piloted twice in Finland, with minor adjustments. In each country, interviews were conducted by one to three junior researchers, Ph.D. candidates, and/or postdoc researchers with experience in qualitative research. A joint training session for researchers was organized in Germany in autumn 2017 to establish a common understanding of the study protocol.

Interviews were conducted from end-2016 to mid-2017. The school staff members were provided information about the research and procedure before signing a written informed consent form (Additional file 4) and participating in the interviews. The interviews lasted approximately between 20 and 60 min. All interviews were audio-recorded, transcribed verbatim, translated into English, and sent to Finland for analysis. Pseudonyms were used in the transcripts, and no information that would clearly identify participants was included. Three transcripts from two schools in Germany were excluded, as we set the minimum number of transcripts per school at three. Interviewers also provided fieldnotes that included reflections on the interviews and descriptions of schools (Additional file 5).

The interviews were analyzed using thematic analysis-a method for developing, analyzing, and interpreting patterns across qualitative data sets. The analytical process consists of systematically coding data to develop themes. The themes constitute the results of the analysis and are understood as patterns of shared meaning across the dataset [29, 30]. The analytical process was iterative: First, AL familiarized herself with the whole dataset to gain a deep overall and context-specific understanding of the data, gaining confidence as an emerging pattern that explain staff’s STP enforcement. Next, AL coded all parts of the data relating to staff confidence. The NVivo program was utilized to organize the coding. AL then re-coded this dataset to analyze the determinants for staff confidence and connections between confidence and enforcement. Finally, AL synthesized the emerging patterns explaining staff confidence in STP enforcement into three themes (here conditions), then compared these themes with the initial dataset on confidence to ensure consistency in the interpretations.

The iterative analysis involved several discussions between AL and MS to reach a consensus on the interpretations. PL and AEK reflected on and agreed to the final considerations. The three conditions (i.e., themes) were detected across the dataset, namely in all seven countries and in all or most schools. Yet, some aspects or nuances within the conditions may be more dominant in some countries or schools than others. If a particular nuance or aspect was mentioned only in one country, it is specifically mentioned in the “Results” section.

## Results

### The challenge of STP enforcement

The staff demonstrate that an ongoing battle for power between staff and students exists. This struggle continuously manifests in students testing the boundaries of school rules, whether breaking a rule on smoking, using phones, wearing hats during class, or littering, and so forth. Testing boundaries was described as *common pupil behavior*. When the staff intervene in students’ rule violations, students often show negative responses, such as complaining, questioning, disregarding staff’s instructions, or saying rude things. These negative responses made enforcement challenging for the staff. *Interviewee: It’s a kind of fear of whether they’re going to talk back—of what they’re going to do. Researcher: Do they talk back? Interviewee: Of course, they’re going to talk back* (NLD2S). Staff’s expectations concerning their ability to cope with students’ negative responses influenced their intervening in rule violations. When the staff felt confident in preventing, diminishing, or handling students’ responses, they were likelier to intervene, whereas when the staff felt uncertain, they were likelier to ignore the violations.

Overall, rule violations were considered most common among students facing a combination of problems related to, for instance, life management skills, academic performance, and smoking addiction. Most staff considered enforcement with these persistent misbehavers challenging as they tended to respond strongly when addressed for violations. *Most of them (students) immediately understand they have done something stupid, but there have also been people who couldn’t care less* (FIN3M). All staff may struggle with enforcement, especially with the persistent misbehavers. However, due to certain characteristics, such as a lack of intrinsic authority, some staff members were generally less confident in addressing students about violations. *Some teachers absolutely have no problem going over and lifting them (students) out of it [i.e., giving it out to them for smoking*] *— your personality is such a huge part of it* (IRL2T1). A lack of professional experience also played a role. Having more professional experience was reflected to decrease a staff’s sensitivity to students’ negative responses and provide better skills to handle the discussion with students. *It is just part of a teacher’s self-development. — If teachers just started teaching, you cannot expect them to function adequately on everything… It just needs time* (NLD1T). Students were stated as being aware of the staff members most likely to ignore violations. Thus, the staff who struggle the most may also encounter more situations that entail addressing students.

### Conditions facilitating staff’s confidence to enforce STPs (see Fig. 1).

**Fig. 1:**
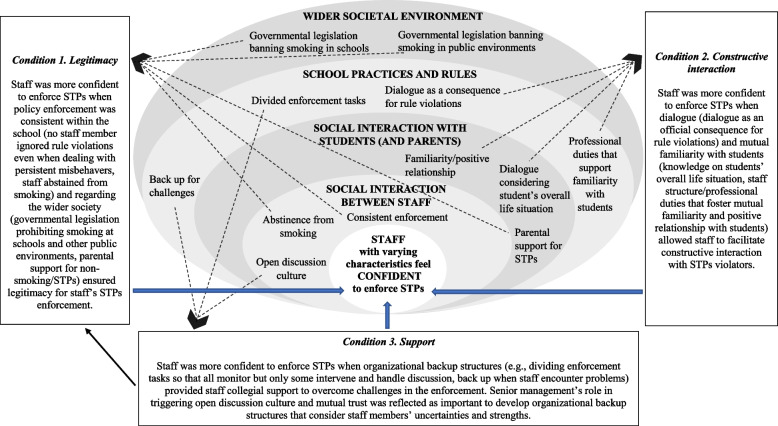
Facilitating conditions for staff’s confidence to enforce school tobacco policies (STPs) structured in line with schools as Social Complex Adaptive Systems (SCAS)

#### Condition 1: Consistent policy enforcement within the school and regarding the wider society ensures legitimacy for staff’s STP enforcement

Governmental legislation sets the minimum requirements for STPs, and thus most of the schools prohibited smoking on the indoor and outdoor premises of the school (Table 1). Clear STPs, underpinned by governmental legislation, supported staff members’ legitimacy for STP enforcement and provided an argument for discussions. *Yes, I have also personally shown [governmental law on STPs] to a student — That pretty much ends the discussion — They know this is how it is* (FIN2M). Also, smoking bans in public places (e.g., cafes, restaurants, shopping malls, public transport) were considered to support students’ acceptance for STPs, whereas negative responses were more common if the school environment was more restrictive than the wider society.

Consistent rule enforcement among all staff members, meaning no one ignored STP violations, was considered critical for a staff’s legitimacy to enforce the rules, as inconsistency allowed students to contest them. *If there are two or three teachers who deviate from that (enforcing the rule), then you can be 100% sure there will be a student who says, ‘Well, this other teacher allows it.’ As a result, you won’t be able to enforce the rule* (NLD1M2). The staff indicated that persistent misbehavers were especially prone to use inconsistencies in enforcement to their advantage. Nevertheless, enforcement also with these students was considered important to ensure consistency. *If you start making exceptions, that’s the end (because then students say) ‘Ah, but you said OK for X.’ Then you can no longer justify it, and the rule isn’t worth anything anymore* (BEL1S).

Staff’s abstinence from smoking was reported important to ensure an individual’s and a whole staff’s legitimacy to enforce STPs and promote a tobacco-free life—an aim often integrated into the STPs. Staff smoking was considered to undermine legitimacy also when it adhered to STPs but was still witnessed by students. *I don’t know what is less educational than when it (smoking) is forbidden inside the school, whatever the space, and then the person (staff member) goes along with the students to smoke outside the gate, in front of the students – the pedagogy of the example, I believe that it is fundamental* (POR2M). Staff’s abstinence from smoking supported staff’s confidence by preventing students’ the opportunity to question the rules: *You don’t give the young boys the opportunity to say, ‘How come you can smoke but I can’t’?* (ITA4T2).

#### Condition 2: Familiarity and dialog allow staff to build up constructive interaction with students during the enforcement

When the staff addressed students for rule violations, they often preferred to build a dialog with students to *change the behavior as opposed to just punishing them* (IRL2T2). Compared to the punitive approach, such as issuing detention, dialog was experienced to decrease students’ need to defend their actions with negative responses and engage adolescents in constructive interaction. Sometimes, dialog was even integrated into the official consequences for rule violations, like the case of Finnish *educational conversations*, which followed a specific procedure: *We fetch them (students), go over the note (STPs violation), and then have a discussion about it. After that, we have the child call home, after which I talk to the guardian* (FIN1T2). Involving parents was stated as necessary as parental approval for smoking was reported to increase students’ opposition to the STPs.

A positive relationship or certain familiarity with students allowed the staff to predict students’ responses and diminish the negative ones by shaping the dialog according to *the hierarchy of the problems in each young person’s life and behavior* (FIN2M). Staff stated that always looking at *the child behind the behavior* is important (NLD2S), which familiarity made easier. In contrary, intervening in unfamiliar students’ rule violations was often experienced as problematic. *I sometimes find it (intervening) a bit difficult, especially if you don’t know the student* (GER3T3).

In countries where supportive staff members, specifically educators, were integrated into the school staff structure, teachers’ and educators’ duties were often differentiated in a way which supported educators’ better familiarity and relationships with students. *They (educators) know many things we (teachers) don’t know about the students. However, it’s also their part (duty) to have a certain familiarity with students, which is a privilege. We aim for that in this school. We aim to have students be able to tell their problems* (BEL1T). Familiarity was said to work both ways, as staff stated that students were less likely to contest staff members with whom they were familiar and, for instance, knew to be strict. *If you’ve had contact with a pupil, they know who you are. They listen better. Otherwise, they look at you and think, ‘Who the hell are you? It’s nice what you’re saying, but I’m turning around now’* (NLD1T).

#### Condition 3: Organizational backup structures provide staff members collegial support to overcome challenges in the STP enforcement

Collegial support was reported especially important for the staff who were naturally less confident in addressing students about rule violations. *The only thing you can do for the people who really find it difficult to do that (address students) is to make sure it becomes easier; the only way to do that is to have a team of teachers who collectively comes into action* (NLD1M1). Senior management was indicated to play a critical role in building mutual trust and open discussion among staff—a requirement for considering the individual uncertainties.

Some schools had considered the individual uncertainties by dividing the enforcement tasks to prevent staff from ignoring the rule violations in challenging situations. For instance, in one school, the staff were not required to address students personally, yet everyone was obligated to monitor and report violations to a specific *list*. Dialog with the students was then conducted by staff with the most jurisdiction (e.g., senior management, section head) or suitable characteristics (e.g., long professional experience) to cope with students’ negative responses. *The educational conversation group takes care of these sorts of behavioral things. An excellent team (two teachers mentioned by name) and I have been doing this for a long time. We all have very long careers and a lot of experience. We know what is going on, and we handle these things. And it does work quite well* (FIN1T2).

Even when addressing students for rule violations was indicated to all staff members, it was common to direct the challenging cases, such as repeated violations or notably rude behavior, to specific staff members—mainly senior management. This organizational backup structure ensured that staff would not be left alone with challenges, encouraging them to also enforce when expecting strong responses from students. *Interviewee: So, in that sense, I feel supported. I know when I find something difficult, I can always rely on someone (e.g., section head) who can deal with the pupils in a different way. Researcher: Do you think that pupils’ knowledge of this increases your authority? Interviewee: Yes, the ‘section head’ is something big for them* (NLD1T). According to staff reflections, the backup structure also quells students’ courage to contest the rules and deter them from progressively bad behavior.

## Discussion

### Summary of the findings

When the staff were confident that they could prevent, diminish, or deal with students’ negative responses, they were likelier to address students on STP violations. However, when the staff were uncertain, they were more prone to ignore violations. This process was not only characteristic of enforcing STPs but also other rules, such as prohibiting mobile phone usage, wearing hats during class, and littering. We discovered three conditions that facilitate staff’s confidence to enforce STPs. Staff seemed more confident when (1) consistency in policy enforcement within school and regarding the wider society ensured staff legitimacy for STP enforcement, (2) mutual familiarity and dialog allowed staff to build up constructive interaction with students during the enforcement, and (3) organizational backup structures provided staff collegial support to overcome challenges in the enforcement. The three conditions are summarized in Figure 1 in line with schools as Social Complex Adaptive Systems (SCAS).   

All staff may feel uncertain about enforcing STPs, especially with persistent misbehavers, yet some staff members were generally less confident in enforcing them. Facilitating conditions strengthened the consistency of staff’s practices by supporting enforcement especially with the persistent misbehavers and among the more uncertain staff members. As the conditions were positioned in somewhat distinct phases of the enforcement continuum, all conditions were important to ensure staff’s confidence: legitimacy (conditions 1) to prevent students’ negative responses, constructive interaction (condition 2) to handle the emerging negative responses, and support (condition 3) to cope with students’ strong responses with help from colleagues.

### Limitations

Data collection included the participation of multiple interviewers to ensure the interviews were held in each country’s native language. Despite the collectively agreed-upon topic guide and joint training session, the level that staff were probed to elaborate on their experiences and perceptions varied across the interviews. Therefore, we could have discovered more details or nuances within the three conditions if all interviewers had comprehensively discussed the meaning of confidence in STP enforcement with the interviewees. Also, translating the data from the original languages to English may have affected the level of details and nuances in some of the transcripts. However, given the large number of interviews from various schools in different countries and the similar patterns detected across the dataset, we believe we produced a consistent and comprehensive understanding of the conditions facilitating a staff’s confidence in enforcing STPs. The trustworthiness of these interpretations is strengthened by close collaboration between two researchers from different countries during the analysis. However, a contribution to the analysis from all participating countries would have further strengthened the considerations.

### Interpretations of the results and practical implications

Aligning with schools as SCAS [24], our findings demonstrate that confidence to enforce STPs among staff with varying characteristics emerges in interactions with other actors, school ethos, organizational practices, and the broader tobacco control environment. Next, we focus on interpreting the results and providing multilevel strategies to strengthen the conditions facilitating staff’s ability to cope with students’ negative responses. Strengthening the conditions may support staff’s enforcement also by diminishing students’ negative reactions, as our findings suggest these conditions increase students’ acceptance of STPs (condition 1), reduce their need to defend themselves with negative responses (condition 2), and decrease their courage to contest (condition 3).

Earlier research has demonstrated that consistent enforcement among the staff is critical to ensure STPs’ effectiveness in decreasing adolescent smoking [8, 9, 13]. Our results show that consistent enforcement was both a cause and an effect of staff’s confidence: Consistent enforcement legitimized staff’s authority to enforce STPs and prevented opportunities for students to question that authority (condition 1). Our findings further demonstrate that all staff members need not have similar enforcement tasks to ensure this consistency. Instead, a staff with varying confidence levels may contribute to consistency in distinct ways. For example, monitoring and reporting the violations instead of ignoring them was critical among the more uncertain staff. In contrast, the more confident the staff could make participation easier for others by handling the discussions or providing backup for the more challenging situations (condition 3).

Aligned with the previous interpretations, we suggest that instead of targeting staff members’ abilities one by one, focusing on strengthening structures and practices that support a staff’s collective ability in STP enforcement (e.g., dividing enforcement tasks, implementing backup structures) could provide a more feasible way to ensure confidence and consistent enforcement. This argument is supported also by the knowledge of self-efficacy. Self-efficacy can be collectively assessed when referring to an individual’s expectations that a group can act effectively together. High perceived self-efficacy is shown to explain participants’ actions regardless of whether the self-efficacy is achieved individually or collectively [31]. Sørlie and Torsheim [32] have also demonstrated the association between increased teacher collective efficacy and decreased student misconduct. Earlier research emphasizes senior management’s important role in building this collective efficacy [33–35], thus, aligning with our findings.

Strengthening adolescents’ social and emotional learning (SEL) to enhance positive social behavior, academic achievements, and less misbehavior [36–39] has stood out in educational discussions during the past years [40, 41], yet school staff’s SEL has remained marginal, despite staff having a fundamental role in modeling these competencies to students. SEL consists of five core social and emotional competencies: self-awareness, self-management, social awareness, relationship skills, and responsible decision-making [42]. According to our findings, staff members differ in their SEL, namely their skills and level of comfort in carrying out dialog and building constructive interaction and positive connections with students. Therefore, strengthening a staff’s SEL could support their ability to build up positive relationships, carry out discussions, and solve conflicts with students, increasing their confidence in addressing students who violate STPs. A recent study by Schreuders et al. [27] demonstrated that students who keep violating STPs often have multiple life challenges, making enforcement with this group a complex balancing act with numerous aims. Our findings add to this determination by showing that strong negative responses among these students challenge a staff’s confidence. Strengthening a staff’s SEL could increase staff’s ability to cope, especially with this group of students. Moreover, modeling a staff’s SEL could particularly benefit these adolescents.

Finally, aligning with earlier research [3, 14, 43–45], we suggest strengthening national tobacco control especially on tobacco-free environments (WHO FCTC Article 8). Our findings demonstrate that staff’s legitimacy for STP enforcement and students’ ability to question the rules strongly intertwine with the broader tobacco control environment and STPs being integrated into the governmental legislation. Strengthening tobacco-free environments nationally could further assist schools in adopting and implementing more comprehensive tobacco policies towards tobacco-free schools. For instance, Hjort, Schreuders, Rasmussen, and Klinker [46] demonstrated in their study that believing that society and workplaces are becoming more smoke-free increased organizational readiness to implement smoke-free school hours (SFSH) in Danish vocational schools. SFSH extends STPs to whole school hours and all tobacco products.

## Conclusions

Our study provided an in-depth understanding of the conditions facilitating staff’s confidence in enforcing STPs. Our results stress the need to implement strategies at multiple levels to support staff’s confidence and consistent STP enforcement towards tobacco-free schools. Our findings emphasize especially the need to consider the social context when implementing STPs. To support staff’s legitimacy for enforcement (condition 1), we suggest reinforcing national tobacco control and school practices that facilitate consistent STP enforcement; to support staff’s ability for constructive interaction with STP violators (condition 2), we suggest strengthening staff’s social and emotional learning; and to support staff’s experience of collegial support (condition 3), we suggest reinforcing staff’s collective ability to cope with students’ negative responses. 

## Supplementary Information

**Additional file 1.** Reporting standards.

**Additional file 2.** Overview of interviewees.

**Additional file 3.** English interview guide.

**Additional file 4.** Information letter and informed consent form.

**Additional file 5.** Field notes.

## Data Availability

The datasets generated and analyzed during the current study are not publicly available due to qualitative data’s highly sensitive and identifiable nature.

## Abbreviations

STPs: School tobacco policies
WHO FCTC: World Health Organization’s Framework Convention of Tobacco Control
SCAS: Social Complex Adaptive Systems
SEL: Social and emotional learning
SFSH: Smoke-free school hours
