# Supplementary material for: Facilitating conditions for staff’s confidence to enforce school tobacco policies: qualitative analysis from seven European cities
Source: Implement Sci Commun. 2022 Oct 22;3:113. doi: 10.1186/s43058-022-00362-7 (PMC9588223; doi:10.1186/s43058-022-00362-7)
Supplement: Supplementary file 1 — Additional file 1. Reporting standards. [file 43058_2022_362_MOESM1_ESM.docx]

**Additional file 1: Reporting Standards**

| **Section** | **SRQR guideline** | **How the manuscript adheres to the reporting standards** |
| --- | --- | --- |
| **TITLE AND ABSTRACT** |  |  |
| **S1 Title** | Concise description of the nature and topic of the study. Identifying the study as qualitative or indicating the approach (e.g., ethnography, grounded theory) or data collection methods (e.g., interview, focus group) is recommended | Title describes the topic of the study concisely and indicates that the study is a “qualitative analysis”. |
| **S2 Abstract** | Summary of key elements of the study using the abstract format of the intended publication; typically includes background, purpose, methods, results, and conclusions | Abstract summarises the key elements of the study divided in ‘Background’, ‘Methods’, ‘Results’, and ‘Conclusions’. |
| **BACKGROUND** |  |  |
| **S3 Problem formulation** | Description and significance of the problem/phenomenon studied; review of relevant theory and empirical work; problem statement | The importance of preventive tobacco policies in decreasing adolescent smoking and exposure to tobacco smoke, the vitality of school staff members consistent enforcement of school tobacco policies (STPs) to ensure their effectiveness, and the limited empirical evidence on determinants for staff’s confidence in enforcing STPs is described. |
| **S4 Purpose or research question** | Purpose of the study and specific objectives or questions | The study responds to the accounts of our previously produced realist review (Linnansaari et al 2019) on the lack of evidence on determinants for staff’s confidence in STPs enforcement by empirically studying the conditions in which staff feel confident in their ability to cope with students’ negative responses in STPs enforcement. The ultimate objective is to provide strategies for schools and decision-makers to support staff members’ consistent STPs enforcement towards tobacco-free schools. Two research questions are defined. |
| **METHODS** |  |  |
| **S5 Qualitative approach and research paradigm** | Qualitative approach (e.g., ethnography, grounded theory, case study, phenomenology, narrative research) and guiding theory if appropriate; identifying the research paradigm (e.g., postpositivist, constructivist/  interpretivist) is also recommended | We analysed cross country school staff members interviews with thematic analysis from Braun & Clarke (2020, 2022) to find themes understood as patterns of shared meaning across the dataset. We explain in the ‘Background’ that we consider schools as Social Complex Adaptive Systems which indicates that we expect staff confidence to emerge in the interplay between various determinants at multiple levels. |
| **S6 Researcher characteristics and reflexivity** | Researchers’ characteristics that may influence the research, including personal attributes, qualifications/experience, relationship with  participants, assumptions, and/or presuppositions; potential or actual interaction between researchers’ characteristics and the research questions, approach, methods, results, and/or transferability | The first author (AL) have a professional teaching background and familiarity with the school context, which assisted in formulating the interview topic guide and conducting the interviews in Finland. In in each country, interviews were conducted by one to three junior researchers, Ph.D. candidates, and/or postdoc researchers to ensure that the interviews were help in country’s native language. All interviewers had earlier experience in qualitative research. To establish a common understanding of the study protocol, a joint training session for researchers was organized. Interviewers provided fieldnotes including reflections on the interviews (Additional file 5). |
| **S7 Context** | Setting/site and salient contextual factor | The study is produced as part of SILNE-R study and employs interview data generated with 81 school staff members in 26 secondary schools in seven European cities: Namur (Belgium), Tampere (Finland), Hanover (Germany), Dublin (Ireland), Latina (Italy), Amersfoort (the Netherlands), and Coimbra (Portugal). The countries included in the study represented great diversity in implementation of national tobacco control policies and strategies. The included cities were all median-sized and close to the national average in terms of socioeconomic level and percentage of non-foreign population.  To ensure rich contextual information, we recruited 3-4 schools in every city that represent different school types (academic – vocational) or were from areas with distinct socioeconomic levels. An overview of student smoking prevalence in each school, schools’ rules on student smoking, and countries’ legislation on smoking in school is presented in Table 1. Also, the recruited interviewees represented varying professional positions, including senior management, teachers, and supportive staff. An overview of the interviewees’ country, school number, professional position, age, sex, and smoking status are presented in Additional file 2. Interviewers provided fieldnotes that included reflections on the interviews and descriptions of schools (Additional file 5).  We consider schools as Social Complex Adaptive Systems. This indicates that in the analysis we aimed to capture determinants at multiple contextual layers (societal, social, school, individual) that may influence the emergence of facilitating conditions for staff’s confidence. |
| **S8 Sampling strategy** | How and why research participants, documents, or events were selected; criteria for deciding when no further sampling was necessary (e.g., sampling saturation) | We recruited three to four varying schools with three to four staff members with varying professional positions (senior management, teaching staff, supportive staff) in every city: Namur (Belgium), Tampere (Finland), Hanover (Germany), Dublin (Ireland), Latina (Italy). These decisions were made in advance of the interviews as we expected to be able to produce comprehensive understanding with 81 cross-country school staff interviews. Within the school, senior management or other school contact person who had the ability to select staff from varying positions and with comprehensive knowledge on STPs enforcement recruited the interviewees. An overview of the interviewees’ country, school number, professional position and age group are presented in Additional file 2. |
| **S9 Ethical issues pertaining to human subjects** | Documentation of approval by an appropriate ethics review board and participant consent, or explanation for lack thereof; other confidentiality and data security issues | Ethical approvals for the research were obtained separately for each city to comply with national standards.  School staff members were provided information about the research and procedure before signing a written informed consent form (Additional file 4) and participating in the interviews. Pseudonyms were used in the transcripts, and no information that would clearly identify participants was included. |
| **S10 Data collection methods** | Types of data collected; details of data collection procedures including  (as appropriate) start and stop dates of data collection and analysis,  iterative process, triangulation of sources/methods, and modification  of procedures in response to evolving study findings | The interviews were conducted from end-2016 to mid-2017. We descibe the iterative analysis process comprehensively in the ’Methods’ section. The process involved several discussions between AL and MS to reach a consensus on the interpretations, that were reflected and approved also by PL and AEK. Authors contributions are specified in the ‘Declarations’. |
| **S11 Data collection instruments and technologies** | Description of instruments (e.g., interview guides, questionnaires)  and devices (e.g., audio recorders) used for data collection; if/how the  instrument(s) changed over the course of the study | The interview topic guide (Additional file 3) was formulated in collaboration with research teams from all countries participating in the SILNE-R research project. The Finnish researchers (AL, PL) coordinated the collaboration. The topic guide was piloted twice in Finland, with minor adjustments. A joint training session for interviewers was organized to establish a common understanding of the study protocol. |
| **S12 Units of study** | Number and relevant characteristics of participants, documents, or events included in the study; level of participation (could be reported in results) | The 81 interviewed staff members from 26 secondary schools in seven European cities represented varying professional positions, including senior management, teachers, and supportive staff (e.g., receptionists, janitors, educators). An overview of the interviewees’ country, school number, professional position and age group are presented in Additional file 2. |
| **S13 Data processing** | Methods for processing data prior to and during analysis, including  transcriptions, data entry, data management and security, verification of data integrity, data coding, and anonymization/deidentification of excerpts | All interviews were audio-recorded, transcribed verbatim, translated into English, and sent to Finland for analysis. Pseudonyms were used in the transcripts, and no information that would clearly identify participants was included. |
| **S14 Data analysis** | Process by which inferences, themes, etc., were identified and  developed, including the researchers involved in data analysis; usually  references a specific paradigm or approach | The iterative analysis process, analytical approach (thematic analysis by Braun & Clarke) and collaboration between researcher is comprehensively described in the ‘Methods’. |
| **S15 Techniques to enhance trustworthiness** | Techniques to enhance trustworthiness and credibility of data analysis  (e.g., member checking, audit trail, triangulation) | The trustworthiness of the interpretations is increased by close collaboration between two researchers from different countries (AL, MS). PL and AEK reflected on and agreed to the final considerations. From each SILNE-R country either work package leader or researcher with key role in school staff interviews accepted submission of the final draft. |
| **RESULTS** |  |  |
| **S16 Synthesis and interpretation** | Main findings (e.g., interpretations, inferences, and themes); might  include development of a theory or model, or integration with prior  research or theory | We discovered three facilitating conditions for school staff members confidence to enforce STPs. The main findings are summarised in Figure 1 and in the beginning of the “Discussion”. |
| **S17 Links to empirical data** | Evidence (e.g., quotes, field notes, text excerpts, photographs) to  substantiate analytic findings | Comprehensive set of illustrative quotes are used to substantiate the findings. |
| **DISCUSSION** |  |  |
| **S18 Integration with prior work, implications, transferability, and contribution(s) to the field** | Short summary of main findings; explanation of how findings and conclusions connect to, support, elaborate on, or challenge conclusions of earlier scholarship; discussion of scope of application/generalizability; identification of unique contribution(s) to scholarship in a discipline or field | Summary of the main findings, interpretations of the results, connections to earlier research and practical implications are discussed. |
| **S19 Limitations** | Trustworthiness and limitations of findings | Limitations and strengths are discussed. |
| **OTHER** |  |  |
| **S20 Conflicts of interests** | Potential sources of influence or perceived influence on study conduct and conclusions; how these were managed | The authors declare there is no conflict of interest. |
| **S21 Funding** | Sources of funding and other support; role of funders in data collection, interpretation, and reporting | The funding sources are specified. The funders had no role in the study design, data collection and analysis, decision to publish, or preparation of the manuscript. |

Modified from O’Brien BC, Harris IB, Beckman TJ, Reed DA, Cook DA. Standards for Reporting Qualitative Research. Academic Medicine. 2014;89(9):1245–51.
