# Supplementary material for: Facilitating conditions for staff’s confidence to enforce school tobacco policies: qualitative analysis from seven European cities
Source: Implement Sci Commun. 2022 Oct 22;3:113. doi: 10.1186/s43058-022-00362-7 (PMC9588223; doi:10.1186/s43058-022-00362-7)
Supplement: Supplementary file 2 — Additional file 2. Overview of interviewees. [file 43058_2022_362_MOESM2_ESM.docx]

**Additional file 2: Overview of interviewees**

| **Country** | **School** | **Code** | **Position** | **Age group** | **Smokers** |
| --- | --- | --- | --- | --- | --- |
| BEL | 1 | BEL1S | Supportive | 40-50 |  |
| BEL | 1 | BEL1T | Teacher | 30-40 |  |
| BEL | 1 | BEL1M | Management | 50-60 |  |
| BEL | 2 | BEL2S | Supportive | 30-40 |  |
| BEL | 2 | BEL2T | Teacher | 40-50 |  |
| BEL | 2 | BEL2M | Management | 40-50 |  |
| BEL | 3 | BEL3S | Supportive | 30-40 |  |
| BEL | 3 | BEL3T | Teacher | 30-40 |  |
| BEL | 3 | BEL3M | Management | 40-50 |  |
| BEL | 4 | BEL4S | Supportive | 30-40 |  |
| BEL | 4 | BEL4T | Teacher | 40-50 |  |
| BEL | 4 | BEL4M | Management | 50-60 |  |
| BEL | 1-4 |  |  |  | 3/12 |
| FIN | 1 | FIN1M | Management | 40-50 |  |
| FIN | 1 | FIN1T1 | Teacher | 30-40 |  |
| FIN | 1 | FIN1T2 | Teacher | 50-60 |  |
| FIN | 2 | FIN2T1 | Teacher | 50-60 |  |
| FIN | 2 | FIN2T2 | Teacher | 30-40 |  |
| FIN | 2 | FIN2M | Management | 50-60 |  |
| FIN | 3 | FIN3T1 | Teacher | 30-40 |  |
| FIN | 3 | FIN3T2 | Teacher | 40-50 |  |
| FIN | 3 | FIN3M | Management | 40-50 |  |
| FIN | 4 | FIN4T1 | Teacher | 40-50 |  |
| FIN | 4 | FIN4T2 | Teacher | 50-60 |  |
| FIN | 4 | FIN4M | Management | 50-60 |  |
| FIN | 1-4 |  |  |  | 1/12 |
| GER | 1 | GER1T1 | Teacher | 50-60 |  |
| GER | 1 | GER1T2 | Teacher | 60-70 |  |
| GER | 1 | GER1T3 | Teacher | 60-70 |  |
| GER | 1 | GER1T4 | Teacher | 40-50 |  |
| GER | 2 | GER2T1 | Teacher | 40-50 |  |
| GER | 2 | GER2T2 | Teacher | 30-40 |  |
| GER | 2 | GER2T3 | Teacher | 30-40 |  |
| GER | 3 | GER3T1 | Teacher | 30-40 |  |
| GER | 3 | GER3T2 | Teacher | 20-30 |  |
| GER | 3 | GER3T3 | Teacher | 30-40 |  |
| GER | 3 | GER3T4 | Teacher | 30-40 |  |
| GER | 1-3 |  |  |  | 2/11 |
| IRL | 1 | IRL1M | Management | 50-60 |  |
| IRL | 1 | IRL1T1 | Teacher | 40-50 |  |
| IRL | 1 | IRL1T2 | Teacher | 50-60 |  |
| IRL | 2 | IRL2T1 | Teacher | 20-30 |  |
| IRL | 2 | IRL2T2 | Teacher | 40-50 |  |
| IRL | 2 | IRL2M | Management | 50-60 |  |
| IRL | 3 | IRL3M | Management | 50-60 |  |
| IRL | 3 | IRL3T | Teacher | 60-70 |  |
| IRL | 3 | IRL3S | Supportive | 40-50 |  |
| IRL | 4 | IRL4S | Supportive | 50-60 |  |
| IRL | 4 | IRL4M1 | Management | 30-40 |  |
| IRL | 4 | IRL4M2 | Management | 60-70 |  |
| IRL | 1-4 |  |  |  | 0/12 |
| ITA | 1 | ITA1S | Supportive | 50-60 |  |
| ITA | 1 | ITA1M | Management | 60-70 |  |
| ITA | 1 | ITA1T | Teacher | 50-60 |  |
| ITA | 2 | ITA2S | Supportive | 50-60 |  |
| ITA | 2 | ITA2M | Management | 40-50 |  |
| ITA | 2 | ITA2T | Teacher | 40-50 |  |
| ITA | 3 | ITA3M | Management | 40-50 |  |
| ITA | 3 | ITA3S | Supportive | 40-50 |  |
| ITA | 3 | ITA3T | Teacher | 50-60 |  |
| ITA | 4 | ITA4T1 | Teacher | 50-60 |  |
| ITA | 4 | ITA4T2 | Teacher | 40-50 |  |
| ITA | 4 | ITA4M | Management | 50-60 |  |
| ITA | 1-4 |  |  |  | 2/12 |
| NLD | 1 | NLD1T | Teacher | 30-40 |  |
| NLD | 1 | NLD1M1 | Management | 50-60 |  |
| NLD | 1 | NLD1S | Supportive | 40-50 |  |
| NLD | 1 | NLD1M2 | Management | 30-40 |  |
| NLD | 2 | NLD2S | Supportive | 60-70 |  |
| NLD | 2 | NLD2M | Management | 50-60 |  |
| NLD | 2 | NLD2T | Teacher | 60-70 |  |
| NLD | 3 | NLD3M | Management | 50-60 |  |
| NLD | 3 | NLD3T | Teacher | 30-40 |  |
| NLD | 3 | NLD3S | Supportive | 50-60 |  |
| NLD | 4 | NLD4T | Teacher | 30-40 |  |
| NLD | 4 | NLD4S | Supportive | 40-50 |  |
| NLD | 4 | NLD4M | Management | 60-70 |  |
| NLD | 1-4 |  |  |  | 2/13 |
| POR | 1 | POR1M1 | Management | 50-60 |  |
| POR | 1 | POR1T | Teacher | 50-60 |  |
| POR | 1 | POR1M2 | Management | 60-70 |  |
| POR | 2 | POR2M | Management | 50-60 |  |
| POR | 2 | POR2T1 | Teacher | 30-40 |  |
| POR | 2 | POR2T2 | Teacher | 30-40 |  |
| POR | 3 | POR3M | Management | 50-60 |  |
| POR | 3 | POR3T1 | Teacher | 50-60 |  |
| POR | 3 | POR3T2 | Teacher | 50-60 |  |
| POR | 1-3 |  |  |  | 5/9 |
