# Supplementary material for: Facilitating conditions for staff’s confidence to enforce school tobacco policies: qualitative analysis from seven European cities
Source: Implement Sci Commun. 2022 Oct 22;3:113. doi: 10.1186/s43058-022-00362-7 (PMC9588223; doi:10.1186/s43058-022-00362-7)
Supplement: Supplementary file 3 — Additional file 3. English interview guide. [file 43058_2022_362_MOESM3_ESM.docx]

**Additional file 3: English interview guide**

**SILNE-R TOPIC GUIDE FOR THE INDIVIDUAL SCHOOL STAFF INTERVIEWS**

**The school staff interviews aims to:**

- Explore how tobacco control policies (restrictions and bans on smoking) and education on tobacco (e.g. interventions, curricular programs, curriculum) are implemented (adopted, enforced, maintained) in schools and how different processes and factors have influenced the implementation.
- Explore staff members` responses and experiences on the implementation of school tobacco-control policies and education on tobacco and how different factors and processes have supported or challenged their activities.

**General information about the interview:**

- Welcome the participant and introduce yourself.
- Give brief information about the SILNE-R project and the aims of the interview: Part of European study including school staff interviews from seven different countries. Exploring staff members` experiences on the implementation of school smoking bans and education on tobacco.
- Describe the practicalities (e.g. timing 45-60 min), explain confidentiality (e.g. anonymity, data protection) and ask if the interviewee agrees with recording.
- Explain the format of the interview (e.g. open questions, exploring staff member`s perspectives on the topics).
- Check if the participant has any questions at this point.
- Ask the interviewee to fill out the informed consent.
- Ask if it is possible to proceed to the interview.
- Start recording.

1. **WARMING UP**

Open the interview by asking briefly some basic information about interviewee`s role and tasks at school and how long she/he has been working in this school.

**Topic 1 Context (e.g. prevalence of smoking, social norms)**

Looking at you school, what kind of role does tobacco have, how visible is smoking and have this changed during the years you have worked in this school?

- What have influenced the change?
  - e.g. overall image of tobacco, changes in law?
- How are the smoking bans or restrictions complied with?

**B) SMOKING BANS**

**Topic 2 Adoption of current smoking ban policies**

Could you describe how long the current bans/restrictions have been in place in your school and did you work in the school when these were adopted and implemented in the first place? Have the bans/restrictions changed during the time you have been working in the school? (If the interviewee has not knowledge on this topic, move to topic no. 3.)

- Why were these specific bans/restrictions implemented?
- Where did the initiative to the implementation come: from the school or outside the school?
- How and by whom was the decision on the implementation made and why were the bans and restrictions formed as they are?
  - e.g. what facilitated or hindered the decision making process leading to implementation of rudimentary vs comprehensive smoking ban/restrictions

**Topic 3 Facilitators and barriers for comprehensive smoking ban (smoking prohibited for students, staff, visitors at all times both inside school buildings and outside premises)**

1. If there is not a comprehensive smoking ban implemented in your school, what are the reasons for this and what would support the adoption and implementation?
2. If there is a comprehensive smoking ban implemented in the school, what were the reasons and possible support for this?

**Topic 4 Responses of staff members**

How have staff members responded to the bans/restrictions on smoking or the changes in them in your school and what could be the possible reasons for these responses?

- Commitment of staff (e.g. principal/vice principal, health promotion coordination team, teaching and non-teaching staff) to the enforcement of tobacco-free environment and what could explain their stance?
- How have the school managed the possible resistance on bans?

**Topic 5 Enforcement practices**

Could you describe the ways in which the restrictions or bans on smoking are enforced in your school and how are these enforcement practices working on a daily basis?

- Possible enforcement practices: communication practices, signs for prohibiting smoking, monitoring during the breaks, consequences for violation, smoking cessation provided etc.
- Which practices are working well and why?
- What kind of challenges are encountered, how have the problems been solved, what is needed to be better tackle the problems?
  - e.g. smoking in the vicinity of school border and entrance, staff smoking
- Why are these specific enforcement practices implemented and not some others?
- Have staff members and/or students or parents participated in planning and developing the enforcement practices?

**Topic 6 Facilitators and challenges for staff members` enforcement activities (decision making and behavior)**

Which factors have supported or challenged staff members` enforcement activities (e.g. does or does not intervene on student smoking) and why have those influenced in these ways?

- e.g. senior management`s support, clearly communicated official stance, feeling of responsibility, knowledge and confidence to take action on student smoking, school characteristics, multiple problems among students, existing workload.

**Topic 7 Maintenance of the policy**

How is the maintenance of the policy managed in your school?

- e.g. monitoring the prevalence of smoking, documenting the violations of the policy, evaluating the progress, updating the policy.

**C) EDUCATION AND TRAINING ON TOBACCO**

**Topic 8 Education practices**

How is tobacco education carried out in your school, what kind of training do adolescent get?

- e.g. Part of specific subjects, curricular programs, interventions on tobacco or other health issues.
  - If multiple, discussed separately.
- Permanent or temporary practices and why?
- Who are carrying out the programs/education?
- If the school does not have any education on tobacco, what are the reasons for this? What could support the adoption and implementation?

**Topic 9 Reasons for adoption and implementation**

Could you describe, why these programs/education, and not some others, have been adopted and implemented in your school?

- How was the decision on the adoption and implementation made?
- Who have been involved in the decision making?

**Topic 10 Best practices on tobacco education**

What kind of education/programs have been working well in your school and what kind have not and what are the possible reasons for this?

- In what ways have staff responded to different kind of education/programs on tobacco and what could be the possible reasons for these responses?
- What kind of education seems appropriate, attractive and effective for students and seems to reach different kind of students, e.g. those who smoke?
- What would be the best way to carry out tobacco education and why?

**Topic 11 Factors influencing staff members readiness to tobacco education**

Which factors have supported or challenged staff members` readiness (commitment, acceptance, motivation) for tobacco education and why?

- e.g. lack of training, existing workload, support from active NGOs.

**D) CLOSURE**

**Topic 12 Further support needed**

What could further support your school to become and enforce more tobacco-free environment?

- e.g. law that enables staff to take tobacco products away from students, closer collaboration with NGOs etc.

**Topic 13 Anything to add**

Is there anything else you would like to discuss on the topic that have not been discussed yet?

**After the interview:**

- Ask interviewee to fill out the short-questionnaire.
- Thank participant for his/her valuable contribution to the project.
- Give interviewee the information letter about SILNE-R project with your contact details.
- Fill out the field notes.
