# Supplementary material for: Facilitating conditions for staff’s confidence to enforce school tobacco policies: qualitative analysis from seven European cities
Source: Implement Sci Commun. 2022 Oct 22;3:113. doi: 10.1186/s43058-022-00362-7 (PMC9588223; doi:10.1186/s43058-022-00362-7)
Supplement: Supplementary file 4 — Additional file 4. Information letter and informed consent form. [file 43058_2022_362_MOESM4_ESM.docx]

**Additional file 4: Information letter and informed consent form**

**INFORMATION LETTER FOR SCHOOL STAFF PARTICIPATING IN INDIVIDUAL INTERVIEWS.**

**SILNE R: Enhancing the effectiveness of strategies to prevent smoking by adolescents**

The SILNE-R project aims to learn, by in-depth comparisons between seven European countries, how strategies to prevent youth smoking could enhance their effectiveness by taking into account the opportunities, barriers and resources present at local levels. Top researchers from different disciplines and different European regions will work together and link up with international research networks concerned with tobacco control or youth health. We will generate the fine-grained evidence that is needed to support decision makers in implementing smoking prevention strategies that are responsive to local conditions, effective in using available resources, and inspired to reduce inequities. The project is funded by European Union`s Horizon 2020 research and innovation programme.

For more information on the project and the progress, please visit the SILNE-R international website.

**SILNE-R, WORK PACKAGE 7: IMPLEMENTING SCHOOL TOBACCO CONTROL POLICIES IN SEVEN EUROPEAN CITIES.**

The general objective of the school-level analysis (WP 7) is to assess which practices, processes, and contextual factors influence the implementation of school tobacco control policies in seven European cities from Belgium, Finland, Germany, Ireland, The Netherlands, Portugal, Italy. The aim is to formulate explanatory theory and context-sensitive guidelines for a comprehensive implementation of school tobacco control policies by combining information gathered in realist-informed systematic review and semi-structured interviews with school staff.

Interviews: we will conduct interviews with three informants among school staff (e.g. principal, vice principal, teachers, non-teaching staff) in four different schools in each of the seven cities. Duration of each interview will be 45-60 minutes and the interviews will be conducted in the school premises. Interviews will be recorded digitally, transcribed verbatim, translated into English and analysed in Finland. Participation in the interviews is voluntary and interviewees can cancel the participation at any time.

The handling of interview data will comply with the Personal Data Protection Act. Only data relevant for the conduct and analysis of the SILNE-R project will be collected. Complete confidentiality of the interview data will apply, and the data processing will conform to the requirements of national and European legislation on data protection. All data obtained will be anonymised and protected from unauthorised access. The name or any identification of the interviewees will not be sent to Finland from other countries. All data will be transferred into password-protected computer systems that are accessible only for particular tasks related to the SILNE-R project.

If you want to ask or add something after the interview, please contact

Your name____________________________Email______________________________

**SILNE-R WORK PACKAGE 7**

**IMPLEMENTING SCHOOL TOBACCO CONTROL POLICIES IN SEVEN EUROPEAN CITIES: SCHOOL STAFF INTERVIEWS**

I have been asked to participate in the above-mentioned scientific study. I have received information about the study and had a chance to ask the researchers questions about the study.

I understand that participation in the study is voluntary and that I have the right not to participate and the right to withdraw my consent at any given time without giving a reason. I also understand that all information given and recorded will be confidential.

**I agree to participate in this study**

Place ________________________________________

____________ ___________________________________________

Date Participant’s signature
