# Supplementary material for: Facilitating conditions for staff’s confidence to enforce school tobacco policies: qualitative analysis from seven European cities
Source: Implement Sci Commun. 2022 Oct 22;3:113. doi: 10.1186/s43058-022-00362-7 (PMC9588223; doi:10.1186/s43058-022-00362-7)
Supplement: Supplementary file 5 — Additional file 5. Field notes. [file 43058_2022_362_MOESM5_ESM.docx]

**Additional file 5: Field notes**

**SILNE-R WP 7 School Staff Interviews: Field Notes**

Researcher’s name: _____________________________

City: ______________________

Interview number (1-3) ___________ in school (A, B, C, D) _________________

Length of Interview (in minutes): _________

School characteristics (e.g. visibility of smoking, size of the school and school grounds, low/high SES, vocational etc.)

__________________________________________________________________________

______________________________________________________________________________________________________________________________________________________

___________________________________________________________________________

Your reflections on the interview.

How did the interview go overall (e.g. atmosphere, interaction)? What went well? What worked less well? Is there anything else WP7 researchers need to know? (e.g. disruptions, problems, negative reactions to questions, etc.)
